# Modeling and Bioinformatics Identify Responders to G-CSF in Patients With Amyotrophic Lateral Sclerosis

**DOI:** 10.3389/fneur.2021.616289

**Published:** 2021-03-18

**Authors:** Siw Johannesen, J. Russell Huie, Bettina Budeus, Sebastian Peters, Anna M. Wirth, Sabine Iberl, Tina Kammermaier, Ines Kobor, Eva Wirkert, Sabrina Küspert, Marlene Tahedl, Jochen Grassinger, Tobias Pukrop, Armin Schneider, Ludwig Aigner, Wilhelm Schulte-Mattler, Gerhard Schuierer, Winfried Koch, Tim-Henrik Bruun, Adam R. Ferguson, Ulrich Bogdahn

**Affiliations:** ^1^Department of Neurology, University Hospital Regensburg, Regensburg, Germany; ^2^Brain and Spinal Cord Injury Center, Weill Institute of Neuroscience, University of California, San Francisco, San Francisco, CA, United States; ^3^Lifedatascience Consulting, Schriesheim, Germany; ^4^Department of Hematology - Internal Medicine III, University Hospital Regensburg, Regensburg, Germany; ^5^Department of Psychiatry and Psychotherapy, University Hospital Regensburg, Regensburg, Germany; ^6^Institute of Molecular Regenerative Medicine, Paracelsus Medical University Salzburg, Salzburg, Austria; ^7^Spinal Cord Injury and Tissue Regeneration Center Salzburg (SCI-TReCS), Paracelsus Medical University, Salzburg, Austria; ^8^Velvio GmbH, Regensburg, Germany; ^9^Center of Neuroradiology, University Hospital Regensburg & District Medical Center Regensburg, Regensburg, Germany; ^10^BDS Koch, Schwetzingen, Germany

**Keywords:** amyotrophic lateral sclerosis, granulocyte-colony stimulating factor (G-CSF), filgrastim, biomarker, modeling, principal component analysis (PCA), cytokines, stem cell

## Abstract

**Objective:** Developing an integrative approach to early treatment response classification using survival modeling and bioinformatics with various biomarkers for early assessment of filgrastim (granulocyte colony stimulating factor) treatment effects in amyotrophic lateral sclerosis (ALS) patients. Filgrastim, a hematopoietic growth factor with excellent safety, routinely applied in oncology and stem cell mobilization, had shown preliminary efficacy in ALS.

**Methods:** We conducted individualized long-term filgrastim treatment in 36 ALS patients. The PRO-ACT database, with outcome data from 23 international clinical ALS trials, served as historical control and mathematical reference for survival modeling. Imaging data as well as cytokine and cellular data from stem cell analysis were processed as biomarkers in a non-linear principal component analysis (NLPCA) to identify individual response.

**Results:** Cox proportional hazard and matched-pair analyses revealed a significant survival benefit for filgrastim-treated patients over PRO-ACT comparators. We generated a model for survival estimation based on patients in the PRO-ACT database and then applied the model to filgrastim-treated patients. Model-identified filgrastim responders displayed less functional decline and impressively longer survival than non-responders. Multimodal biomarkers were then analyzed by PCA in the context of model-defined treatment response, allowing identification of subsequent treatment response as early as within 3 months of therapy. Strong treatment response with a *median* survival of 3.8 years after start of therapy was associated with younger age, increased hematopoietic stem cell mobilization, less aggressive inflammatory cytokine plasma profiles, and preserved pattern of fractional anisotropy as determined by magnetic resonance diffusion tensor imaging (DTI-MRI).

**Conclusion:** Long-term filgrastim is safe, is well-tolerated, and has significant positive effects on disease progression and survival in a small cohort of ALS patients. Developing and applying a model-based biomarker response classification allows use of multimodal biomarker patterns in full potential. This can identify strong individual treatment responders (here: filgrastim) at a very early stage of therapy and may pave the way to an effective individualized treatment option.

## Introduction

Amyotrophic lateral sclerosis (ALS) is a severe neurodegenerative disorder with a median survival time of 15.8 months from diagnosis (1). Disease etiology and pathophysiology are multilayered, genetic factors, and inflammation, both systemic and within CNS, currently receive maximum attention (2).

Filgrastim (granulocyte colony-stimulating factor, G-CSF) is a safe and widely used FDA-approved drug to mobilize hematopoietic stem cells. Filgrastim is also a neuronal growth factor with neuroprotective and regenerative properties (3, 4), enhancing immunocompetence (5), reducing neuroinflammation (4), increasing motoneuron functional activity (4, 6), and improving motor function and survival in ALS mouse models (4, 7). Since its approval in the early 1990s, filgrastim has been applied in millions of patients and healthy donors. It is an established and well-tolerated clinical compound, so far also in some preliminary studies with ALS patients (4, 8–12).

We provided individual filgrastim treatment to ALS patients in our ALS-outpatient clinic. Filgrastim was applied subcutaneously in a cyclic fashion with individually adjusted dosing. Monthly safety monitoring included assessment of functional decline and survival, as well as gathering of serum retain samples. MRI scans were performed every 3 months. Patient safety and biomarker data from our population have been analyzed earlier (8–10, 13), because lack of a control group assessment of potential objective efficacy of filgrastim in ALS was not possible.

Subsequently, we compared survival and functional decline of filgrastim-treated ALS patients to the largest publicly available collection of ALS patient data from international clinical trials, the PRO-ACT database (Pooled Resource Open-Access ALS Clinical Trials Database)^1^. By comparing filgrastim patient data to PRO-ACT and subgroups of PRO-ACT, we explored and estimated a potential benefit from filgrastim treatment, both for disease progression and for survival.

Biomarkers for disease progression and treatment response are essential in ALS. In view of the heterogeneity concerning disease etiology, pathophysiology, and clinical phenotype, it seems unlikely that single biomarkers could provide this information in total. Rather, an integrated observation of panel biomarkers within different biological systems over time could be useful in the understanding of such a complex disease—and why ALS patients respond so differently to a given treatment. We generated models for survival prediction to identify ALS patients, who might benefit most from this new long-term treatment approach, and assessed development in different biomarker domains [hematological parameters, stem cells, cytokines, and structural changes in brain architecture (Magnetic Resonance Diffusion Tensor Imaging, DTI-MRI)] in responding and non-responding patients over time. Non-linear principal component analysis (NLPCA) has been successfully applied in CNS disorders such as traumatic brain injury (14). NLPCA allows inclusion of different biomarker domains into one systematic analysis. It is a robust descriptive tool to reduce the dimensionality of large numbers of variables, while also handling various data types (ordinal, scalar, etc.) and missing values. We used this novel and data-driven approach to a multitude of biomarkers to estimate treatment effects and individual patients' prognosis.

## Methods

### Description of Filgrastim-Treated Patients and Intervention

Treatment with subcutaneous filgrastim was offered since January 2010 to 36 patients with definite or probable sporadic ALS according to the revised El Escorial criteria (15) after written informed consent. All patients received standard care and riluzole 100 mg/day. The ethics committee of the University of Regensburg approved a retrospective analysis (ethics approval: 15-101-0106 and 14-101-0011). The data were analyzed as of August 31st, 2017. Individual dosing and application were defined upon initiation and adapted over time with the general intention of achieving sufficient hematopoietic stem cell mobilization to peripheral blood (Supplementary Figure 1, Table 1) (Further information in Supplementary Material).

**Table 1:** Baseline characteristics and key survival data of filgrastim-treated patients and the PRO-ACT subgroups.

	**Filgrastim**	**PRO-ACT riluzole and placebo**	**PRO-ACT riluzole and investigational drug**	**PRO-ACT no riluzole, or unknown**
**Number of patients**	**36**	**708**	**2,044**	**1,829**
**Age** (mean)	**51.9 years**	**58.4 years**	**58.8 years**	**58.8 years**
95% confidence interval	48.4–55.4	57.5–59.3	58.2–59.5	50.0–60.0
Pat. with measurements	36	542	951	1624
**ALSFRS-R** (mean)	**37.6/48**	**36.9/48**	**37.0/48**	**36.7/48**
95% confidence interval	35.7–39.5	36.4–37.3	36.6–37.4	36.4–37.0
Pat. with measurements	36	707	825	1513
**Latency between onset and treatment**	**595 days**	**557 days**	**576 days**	**605 days**
95% confidence interval	476.7–713.4	530–583.4	551.3–600.8	586.5–622.8
Pat. with measurements	36	707	823	1532
**Sex** (% female)	**30.6%**	**39.4%**	**42.8%**	**40.3%**
**Onset of symptoms** (n)	**30/36 limb**	**425/708 limb**	**521/2,044 limb**	**504/1,829 limb**
Pat. with measurements	36 (100%)	614 (87%)	699 (34%)	775 (42%)
**Percent from “new” studies**	**100%**	**79.9%**	**24.8%**	**33.5%**
**Treatment duration** (mean)	**17.3 months** (range 2.7–78.8)	n.a.	n.a.	n.a.
**Treatment response**	**15/15/6** (yes/no/n.a.)	n.a.	n.a.	n.a.
**Survival in months** (median)	**19.58**	**13.07**	**10.12**	**11.33**
95% confidence interval	12.39–35.48	12.39–13.76	9.59–10.48	10.97–11.73
**Patients with right-censored survival times**	**11**	**266**	**272**	**424**

*ALSFRS-R data were imputed from ALSFRS data if necessary. “New” studies refer to trials that were added in the latest version of PRO-ACT and/or had the revised score ALSFRS-R. The overall survival dataset (N = 4,617) includes the 36 filgrastim-treated patients and the PRO-ACT survival dataset comprising the three treatment groups. All survival data defined from start of treatment*.

### Description of PRO-ACT, Statistical Analysis Plan, and Cohort Comparability

The survival and functional decline control dataset was based on the latest version of PRO-ACT (release January 2016)^1^, containing 10,723 fully de-identified clinical ALS patient records from 23 phase II/ III trials. The scientific validity of this dataset has been shown in numerous publications (14, 16–21). Information on handling of missing data and problematic data issues is described in the Supplementary Section. We generated a statistical analysis plan (finalized April 5th, 2018) to explore and estimate potential benefit in disease progression and survival of filgrastim-treated patients in comparison to PRO-ACT. After testing the robustness of the treatment effects, a model for survival prediction helped to identify individual response. Within PRO-ACT, the subgroup of riluzole and placebo-treated patients, termed “rp-PRO-ACT,” showed the highest survival compared to all other subgroups and was considered as the best comparator to filgrastim. The comparability at baseline in the filgrastim-treated patients and the PRO-ACT database was analyzed by standard descriptive statistical methods (Further information in Supplementary Material).

### Survival Analyses

Survival analyses were conducted on a final PRO-ACT dataset of 4,617 patients as defined in the Supplementary Material. All survival data were defined from start of treatment. For statistical adjustments, the following variables were considered: age, sex, ALSFRS-R, site of onset (limb or bulbar), treatment latency, and riluzole use. “Databases” describes filgrastim or PRO-ACT groups. In addition to classical proportional hazards models, the additional accelerated failure time (AFT) analysis in patient subgroups describes hazard of covariates upon acceleration/deceleration in the disease course by an event time ratio (ETR). Further, a matched-pair approach was applied on PRO-ACT and filgrastim patients (Further information in Supplementary Material).

### Analysis of Functional Decline

The database contained 60,928 ALSFRS-R measurements of 6,599 PRO-ACT and 36 filgrastim patients. Multiple linear regression models of ALSFRS-R included the following independent variables: database (filgrastim vs. PRO-ACT subgroups), time of ALSFRS-R measurement modeled as three-knot spline, database*month (representing the interaction effect), ALSFRS-R score and age at treatment initiation, treatment latency (onset delta), and sex; the indicator variable for ALSFRS-R score imputation at baseline (ALS BL Imp Ind) was included where it had significant influence. The interaction term “database*month” quantified the difference in steepness of ALSFRS-R decline between groups. The analyses were focused mainly on the first 6 months of treatment. The estimated mean ALSFRS-R course was compared in patient subgroups. An extended model, including three-way interactions (database*month*onset delta), tested dependency on treatment latency (Further information in Supplementary Material).

### Models to Estimate Treatment Effect on Survival Time

To allow estimation of survival times in individual patients, a general linear model with exponential distribution and a reciprocal link function was built on deceased rp-PRO-ACT patients. Variables taken were ALSFRS-R slope (robust estimate), age, and treatment latency. The model-predicted individual survival was correlated and compared to the observed survival within this subgroup. Next, the model was applied to all PRO-ACT and filgrastim-treated patients (including censored patients). Subsequently, hypothetical survival was compared to observed survival. Filgrastim treatment response was derived from the difference between individual model-predicted and observed survival, a Kaplan–Meyer analysis for both groups was conducted. To address the question, if ALSFRS-R time courses differ dependent on filgrastim response, treatment response was added in the mixed-effect model for estimation of functional decline.

### Biomarker Signature for Individualized Treatment Response

We assessed cytokines at baseline and 3, 6, 9, and 12 months by multiplex electrochemiluminescence. Hematological parameters were assessed at the same timepoints. CD34^**+**^ and CD34^**+**^CD38^**−**^ hematopoietic stem and progenitor cells (HSPC) were analyzed in peripheral blood by flow cytometry as previously described (10). Structural MRI was conducted at two 1.5-Tesla scanners. We used the model-generated filgrastim response groups and evaluated blood cytokines by an area under the curve (AUC) approach. We used the baseline value of the analyzed biomarker as bottom border and a line connecting the measured values at 3, 6, and 9 months as upper border.

Several biomarkers with a multitude of dimensionality were detected in filgrastim treated patients over time. Non-linear principal component analyses were applied in the evaluation of hematological parameters, stem cells, cytokines, and structural changes in brain architecture (diffusion tensor imaging, DTI-MRI) as a robust descriptive tool to reduce the dimensionality of large numbers of variables, handling various data types (ordinal, scalar, etc.) and missing values (14). A non-linear principal component analysis (NLPCA) was used to determine covariance among variables within each biomarker package. NLPCA was performed for each biomarker package separately at three time points: all available measures up to 3 months, up to 6 months, and up to 12 months. The goal of these analyses is to (1) determine which variables at each time point are most highly correlated with the variance explained by a particular principal component, (2) identify the emergent “identity” of each principal component, and (3) use the normalized principal component scores (PC scores) to run specific hypothesis tests of group differences. This approach allowed us to reduce a large number of variables into a single composite outcome score for each PC and then perform a single hypothesis test, rather than run multiple tests on individual outcome measures, increasing our probability of committing a Type I error. For the following analyses, only those variables that were over an absolute loading threshold of 0.5 are shown; this thresholding step allows domain experts to focus only on those variables that are most strongly correlated with the variance explained by their respective PCs, in order to best identify the identity of that PC. The statistical significance level for all analyses was set to α = 0.05. Data for DTI–ROI determinations, cell mobilization, and hematology were treated accordingly.

A linear mixed model was conducted, with the previously determined responder categories serving as independent variables (Further information in Supplementary Material).

## Results

### Patient Cohorts

Thirty six sporadic ALS patients (25 male, 28 limb onset, eight bulbar onset) received filgrastim in addition to riluzole (Table 1). Mean age at treatment initiation was 51.9 years, mean latency between symptom onset and treatment initiation was 595 days, and mean ALSFRS-R was 37.6/48. Dose and application modes were individualized, and detailed description may be seen in Supplementary Figure 1. The mean dose of 480 Mio IU/month (range 90 to 2,160 Mio IU/month) was applied subcutaneously, mainly as 5-day treatment block once or twice a month, or continuously on single days (mostly every second day). The median duration of treatment was 13.8 months (mean 17.3 months; range from 2.7 to 78.8 months). Filgrastim was well tolerated, and safe, minor adverse events were mild to moderate bone pain after injections, and—as expected—leukocytosis. One patient developed a possible drug-related intolerance or mild allergic reaction after 39 months of filgrastim treatment (Supplementary Figure 1). Baseline characteristics in comparison to PRO-ACT are provided in detail in Table 1. Adjustments are detailed in Supplementary Material.

### Survival and Progression

**Figure 1:**
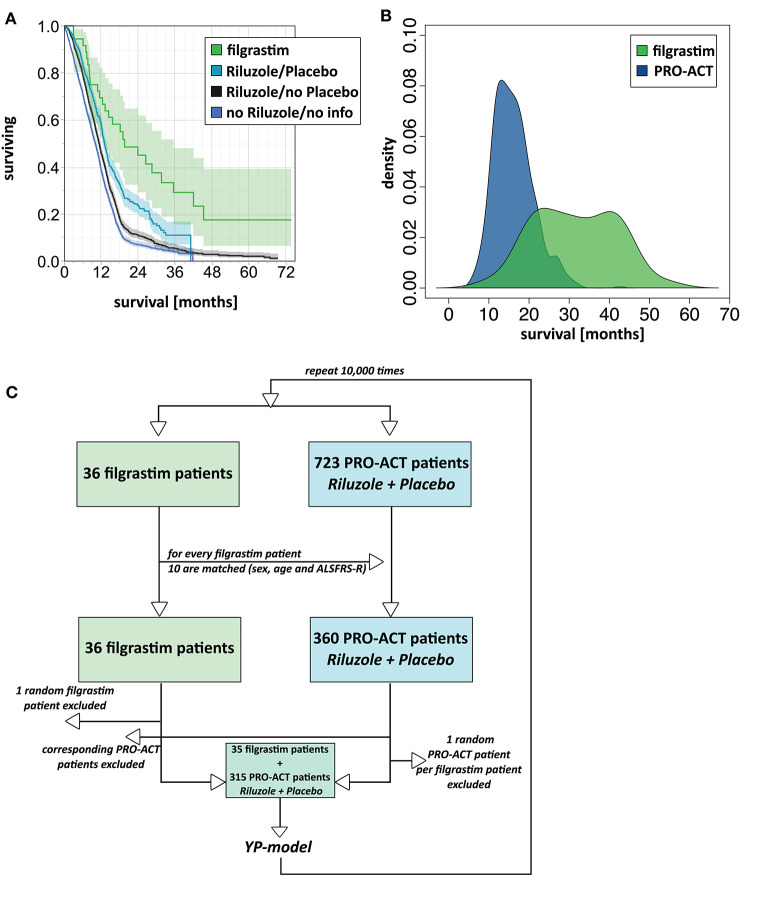
Survival analyses. **(A)** Unadjusted survival analysis comparing filgrastim with PRO-ACT groups. Kaplan–Meier survival plot with shaded pointwise 95% CIs, by study group. Green: filgrastim group; turquoise: rp-PRO-ACT group (riluzole- and placebo-treated PRO-ACT); black: riluzole, no placebo-treated PRO-ACT; blue: PRO-ACT group not treated with riluzole/or not known treatment. All data defined from start of treatment. **(B)** AFT model for survival analysis. Density plot of predicted survival times from the accelerated failure time (AFT) model in rp-PRO-ACT subgroup (blue) and filgrastim (green). Survival times are given by the time to event. The HR for PRO-ACT patients over filgrastim patients was 3.5, the event-time ratio for filgrastim was 0.55. **(C)** Model scheme of the matching plus bootstrapping approach in matched-pair analysis. For the matching with bootstrapping approach, the matching algorithm was first tested in a reduced number of 10 draws and by comparison of age and ALSFRS-R in the filgrastim patients and their 10 matching rp-PRO-ACT patients. Adequate matching was challenged by visual proof and test for equivalence (TOST). Survival comparison: From the 10 respective rp-PRO-ACT matching counterparts of each filgrastim patient, 9 were randomly included to a survival analysis together with the filgrastim patients, and survival curves were analyzed by the YP model by Yang and Zhou (22) (Supplementary Material).

The filgrastim group showed a significant median survival benefit (19.6 months, 95% CI 12.4–35.5) from the first year onward compared to all PRO-ACT patients (11.1 months, 95% CI 10.7–11.3, *p* < 0.0001 by log-rank test) and the subgroup of rp-PRO-ACT patients (13.1 months, 95% CI 12.4–13.8, *p* = 0.0029 by log-rank test; overall test between all groups: *p* < 0.0001, log-rank and Wilcoxon test; Figure 1A, Table 1).

In 1,544 PRO-ACT and filgrastim patients, *age, sex, baseline ALSFRS-R, site of onset, treatment latency*, and *riluzole use* were used as covariates for adjusted parametric survival analyses. Contributions of all covariates were significant, except for riluzole use. The Cox proportional-hazard ratio for all PRO-ACT patients over filgrastim was 2.44 and 0.41, respectively (*p* < 0.0001, Supplementary Figure 2); for rp-PRO-ACT patients (*n* = 499), the Cox proportional-hazard ratio was 1.93 and 0.52, respectively (*p* = 0.0052; Table 2). The estimated significant survival benefit was smaller in *male* patients, in *bulbar onset*, in *higher age*, in *lower baseline ALSFRS-R*, and in *longer treatment latency*. In the AFT model, an HR of 2.2 and an ETR 0.63 were obtained by comparing the filgrastim cohort to rp-PRO-ACT patients (increase in median survival by 1.6-fold) (Figure 1B). In a “Matched Pairs” approach (Figure 1C), the estimated mean survival in filgrastim-treated patients was significantly longer (596 days), compared to the PRO-ACT-matched patients (373 days; survival given as mean of the medians in all 100,000 draws; all *p* < 0.001 by YP test; Supplementary Figure 3). Comparison of filgrastim (596 days) with the matched patients from the rp-PRO-ACT subgroup again revealed a significant difference with survival at 403 days in this group (median *p*-value of 0.005, range of *p*-values: 0.0001 to 0.07, by YP test; Supplementary Figure 3).

**Table 2:** Adjusted Cox proportional hazard fit and parametric survival analyses with available riluzole- and placebo-treated PRO-ACT patients.

**Variable**	**Cox PH model**		**Parametric model**	
	**Wald test**		**Wald test**	
	**Wald chi**	***p*-value**	**Wald chi**	***p*-value**
	**square**		**square**	
ALSFRS-R at baseline	44.88	<0.001	43.07	<0.001
Age	41.24	<0.001	47.87	<0.001
Database	7.80	0.0052	13.09	0.0003
Sex	5.59	0.0180	6.64	0.0100
Treatment latency	5.24	0.0220	4.77	0.0289
Site of onset	9.52	0.0231	9.99	0.0186

*Statistical significance of survival contribution of a factor is given by its p-value. The significant term “Database” refers to filgrastim or rp-PRO-ACT (riluzole- and placebo-treated PRO-ACT) patients and indicates treatment effect. Number of events 395; number of censorings 140; total number 535. AIC (4,214.85) and BIC (4,248.83) indicate the Akaike and Bayesian information criterion, respectively. “ALSFRS-R at baseline” indicates ALSFRS-R at first visit and was imputed from ALSFRS where necessary. “Treatment latency” gives the latency in days between onset of disease and treatment start*.

**Figure 2:**
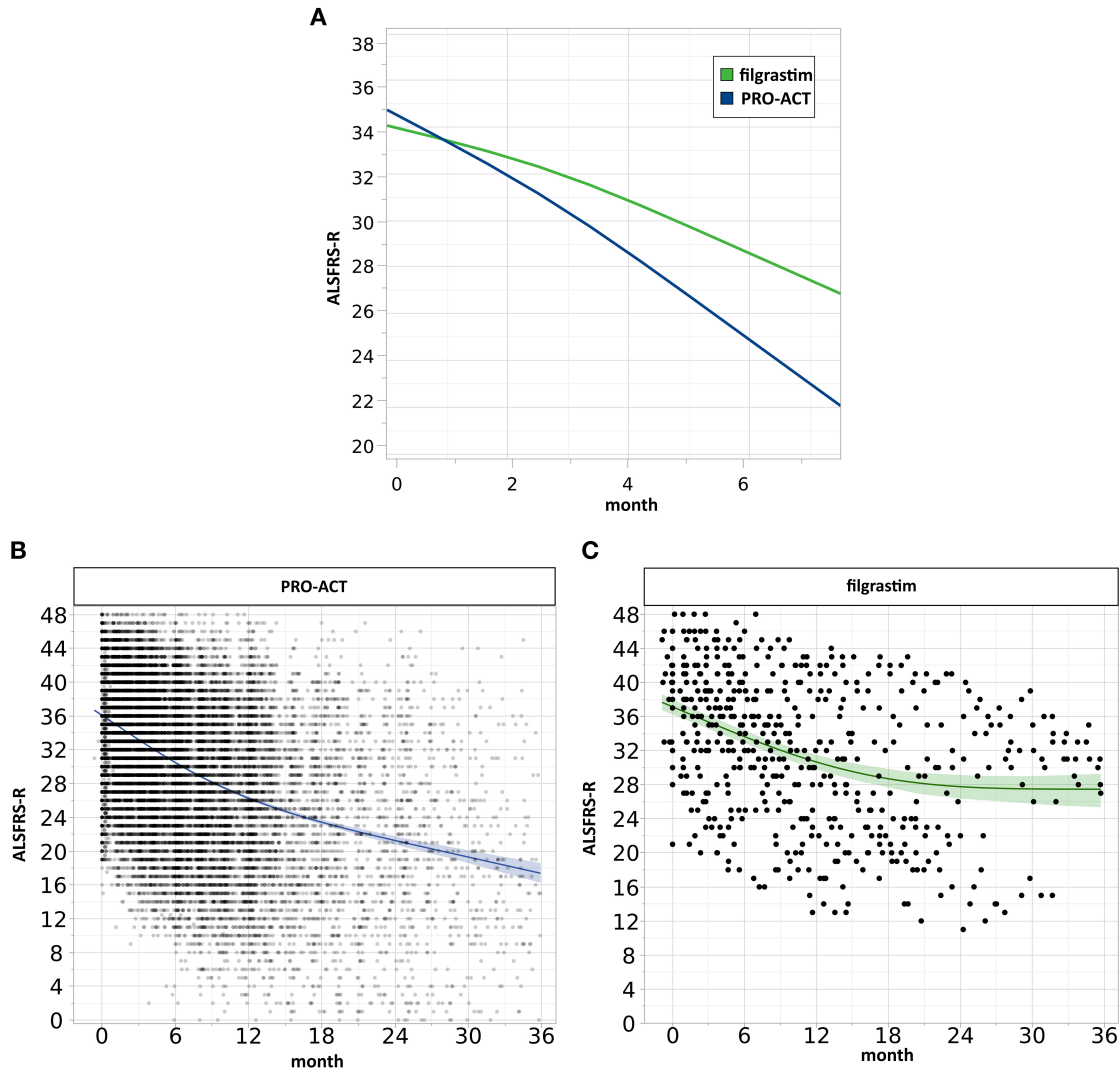
Analysis of clinical progression. **(A)** Estimated ALSFRS-R courses. The interaction profile of estimated ALSFRS-R score time courses of filgrastim- (green, *N* = 36) and riluzole-treated PRO-ACT patients from new studies only (blue, *N* = 6,927) with treatment latency of 10 months includes measurements up to 6 months. The model-estimated decline in filgrastim-treated patients was flatter (interaction term *p* = 0.0002), and the estimated difference between filgrastim and PRO-ACT after 6 months was 3.7 ALSFRS-R score points. **(B,C)** Visualization of individual ALSFRS-R data points over 36 months. Individual ALSFRS-R scores (dots) over 36 months. **(B)** (blue): *N* = 6,927 riluzole receiving PRO-ACT patients, **(C)** (green): *N* = 36 filgrastim-treated patients. Individual data points and smoothing splines visualized the ALSFRS-R scores with 95% CIs. Filgrastim patients stabilize at around 28 ALSFRS-R points; however, already here 2 populations are recognizable. PRO-ACT patients stabilize at around 17 ALSFRS-R points.

Mixed effect modeling of ALSFRS-R functional decline over 6 months was compared in filgrastim- and riluzole-treated PRO-ACT patients (6,927 measurements). The interaction term database*month (*p* < 0.0001) indicates a treatment difference in functional decline *evolving linearly over time* (Supplementary Figure 4). Using a more complex three-way interaction term model, we found an inverse relation between the estimated difference in ALSFRS-R scores between filgrastim and PRO-ACT groups, and treatment latency. In patients with treatment latency of 10 months, the model-estimated difference between filgrastim and patients from new PRO-ACT studies differed by 3.7 ALSFRS-R score points in favor of filgrastim (*p* = 0.0002, interaction term; Figure 2A, Supplementary Figure 6). Individual data points and smoothing splines also visualized the ALSFRS-R scores up to 6 and 36 months in both groups (Figures 2B,C, Supplementary Figure 5).

### Models to Estimate Individual Patient Treatment Effects

**Figure 3:**
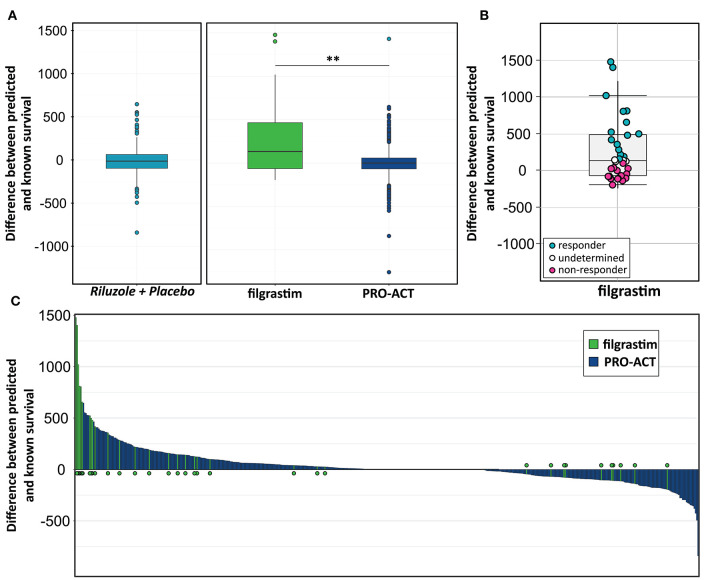
Modeling for survival estimation. **(A)** Model for survival estimation. A general linear model for individual patient survival prediction based on patient variables (calculated ALSFRS-R slope, age, and treatment latency) was built in 447 deceased rp-PRO-ACT subgroup patients. The resulting model-predicted patient survival correlated with the observed survival within this subgroup (*p* < 0.0001; first, turquoise graph). This observation remained identical when applying the model to all PRO-ACT patients (blue graph). The observed survival in filgrastim patients (green graph) was significantly longer than in all PRO-ACT patients and in rp-PRO-ACT subgroup (*p* < 0.001, Wilcoxon test). **(B)** Difference between observed and model-predicted survival. The differences between observed and model-predicted survival in individual filgrastim patients are given by individual dots. Turquoise dots: responders (*n* = 15); pink dots: non-responder (*n* = 15); white dots: non-assignable patients (*n* = 6). **(C)** Waterfall plot for difference between observed to model-predicted survival. Waterfall plot illustration of the difference between observed and model-predicted survival in filgrastim patients (green lines, *N* = 36) and PRO-ACT patients (blue lines, *N* = 6,927), where positive values indicate longer than predicted individual survival. **p < 0.001.

**Figure 4:**
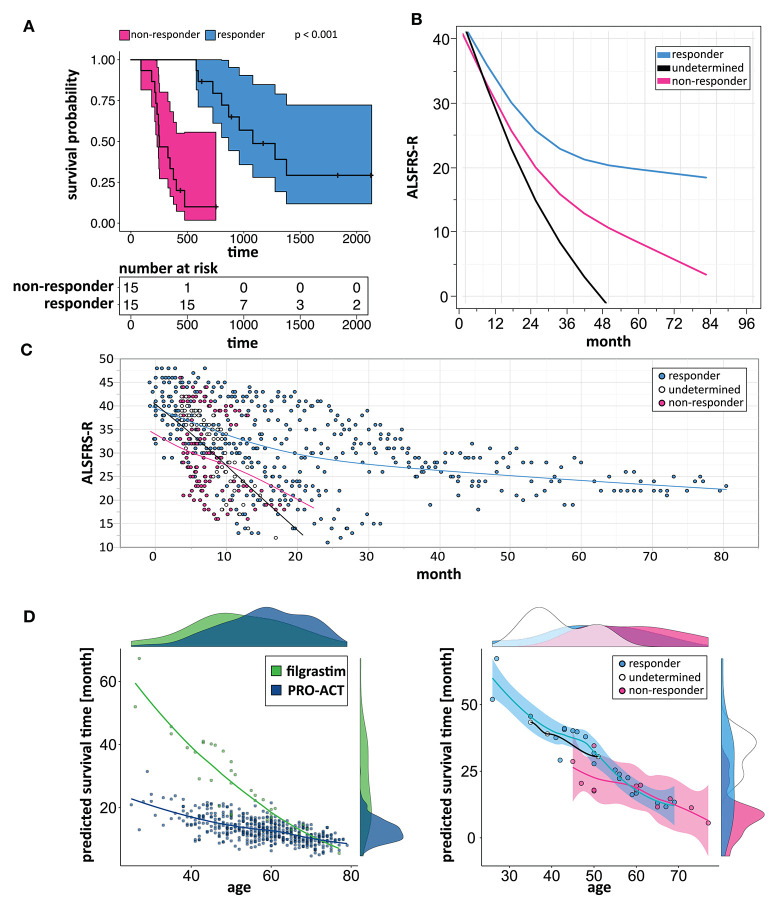
Modeling to identify individual patient response. **(A)** Kaplan–Meier plots of responding and non-responding patients from Figure 3B. Kaplan–Meier survival plot of responding (turquoise, *N* = 15) and non-responding (pink, *N* = 15) filgrastim patients with shaded pointwise 95% CIs. Model-identified responders had a significant survival benefit over non-responders with 1,378 vs. 337.5 days median survival (*p* < 0.0001, log-rank test). **(B)** Estimated ALSFRS-R courses in different filgrastim response groups. Interaction profile of estimated ALSFRS-R score time courses in the different filgrastim response groups with responding patients given by turquoise color, non-responding by pink, and non-assignable patients indicated by black color. A slowing of functional decline is impressive in responders. An increasing difference in functional decline—depending on individual filgrastim response—was revealed by the significant interaction term month*responder group (*p* < 0.0001). **(C)** ALSFRS-R scores over time in different filgrastim response groups. ALSFRS-R scores over time in individual filgrastim-treated patients with responding patients given by turquoise color, non-responding by pink, and non-assignable patients by white color. The interaction term from **(B)** is valid here too. **(D)** Individual model-predicted survival times depending on age. The individual predicted patient survival (dots) dependent on age is illustrated in filgrastim (green) and rp-PRO-ACT patients (blue, both in first graph), and within filgrastim-treated patients (responding: turquoise, non-responding: pink, and non-assignable patients: white color; all second graph). The smooth curves of the second graph represent the model-predicted survival times (y-axis), dependent on age (x-axis). The first graph indicates that especially younger ALS patients benefit from filgrastim treatment but that a treatment benefit might be expected up to ~60 years of age. The second graph illustrates the filgrastim response groups and supports this assumption but also shows that the filgrastim-responding group contains patients up to ~70 years of age; the response assessment of these patients can only be fully understood as additional covariates beyond age have been taken into account.

We build a general linear model for individual patient survival prediction on influential patient variables (calculated ALSFRS-R slope, age, and treatment latency) derived from 447 deceased patients in the rp-PRO-ACT subgroup. The resulting model-predicted patient survival correlated with the observed survival within this subgroup (*p* < 0.0001) with a difference near zero. This remained when applying the model to all PRO-ACT patients. The difference between observed and model-predicted survival was significantly greater in filgrastim patients than in all PRO-ACT patients and in the rp-PRO-ACT subgroup (*p* < 0.001, Wilcoxon test, Figure 3A). A Waterfall plot illustrates the distribution of patients with longer (“responders” = 15) over shorter (“non-responders” = 15) than model-predicted survival in individual filgrastim-treated patients (Figure 3B). Patients with negligible difference were classified as “non-assignable” (*n* = 6, Figure 3C). Model-identified responders had a significant survival benefit over non-responders with 1,378 vs. 337.5 days median survival after start of treatment (*p* < 0.0001, log-rank test; Figure 4A). This was reflected by slowing of functional decline, when adapting the mixed effect model and replacing the database variable by responder group (Supplementary Figure 7) in slope analysis. An increasing difference in functional decline—depending on individual filgrastim response—was revealed by the significant interaction term month*responder group (*p* < 0.0001, Figure 4B) and the plotted individual ALSFRS-R data points (Figure 4C). As age was found to be a strong covariate in survival, the parametric survival model, extended by the interaction term database*age, confirmed this dependency between age and treatment (Supplementary Figure 8). When correlating model-predicted survival times with patient age in the context of database (filgrastim vs. PRO-ACT), as well as in context of filgrastim response (responder vs. non-responder), a strong association of filgrastim-induced survival response with age was evidenced (Figure 4D): younger patients up to 60 years obviously had a much higher chance for this type of response—but interestingly some older patients were also responders.

### Biomarker Signature for Individualized Treatment Response

**Figure 5:**
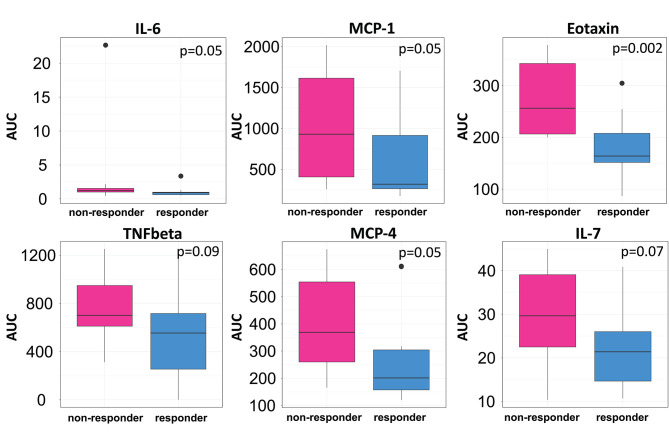
Cytokine levels in filgrastim response groups. Differences in serum cytokine levels between responding (Turquoise) and non-responding patients (Pink; *N* = 15 in each group) were assessed by area under the curve (AUC) over the first 9 months of filgrastim treatment (IL 6 *p* = 0.05, MCP 1 *p* = 0.05, eotaxin *p* = 0.002, TNF-beta *p* = 0.09, MCP 4 *p* = 0.05, IL 7 *p* = 0.07). Initial values obtained before first filgrastim treatment were used as baseline for the AUC calculation. Shown are only items with *p* < 0.1.

**Figure 6:**
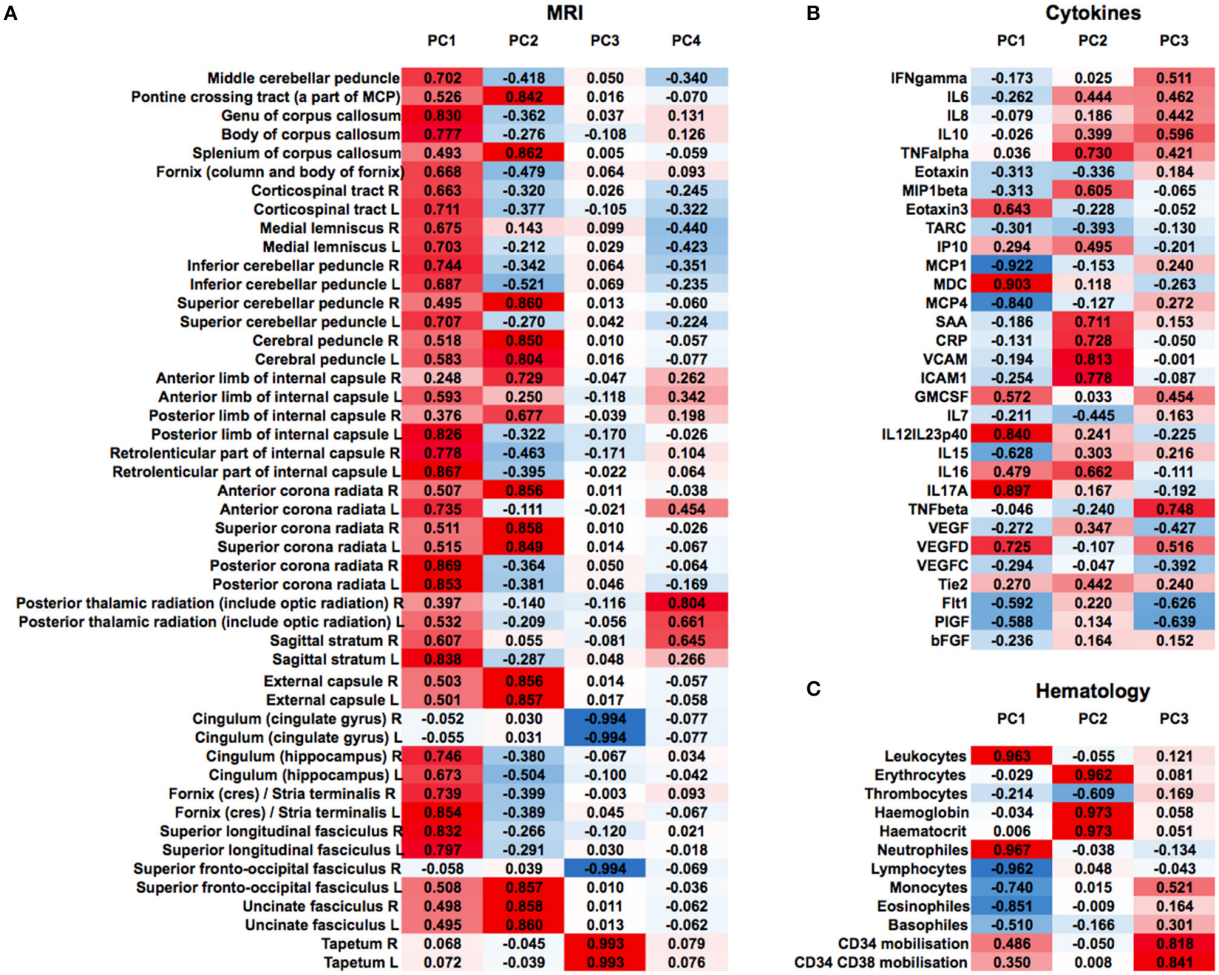
Non-selected PCA at 12 months of filgrastim treatment. Unselected non-linear principal component analyses (PCA) of neuroimaging data [**(A)**, first heat plot: fractional anisotropy (FA) in cerebral MRI DTI], cytokines and growth factors [**(B)**, second heat plot], and hematology parameters [**(C)**, third heat plot] at 12 months of filgrastim treatment. For single items (e.g., specific cytokine), the loading is given in absolute numbers and used for ranking; red color indicates positive magnitude of loading, blue negative magnitude of loading.

**Figure 7:**
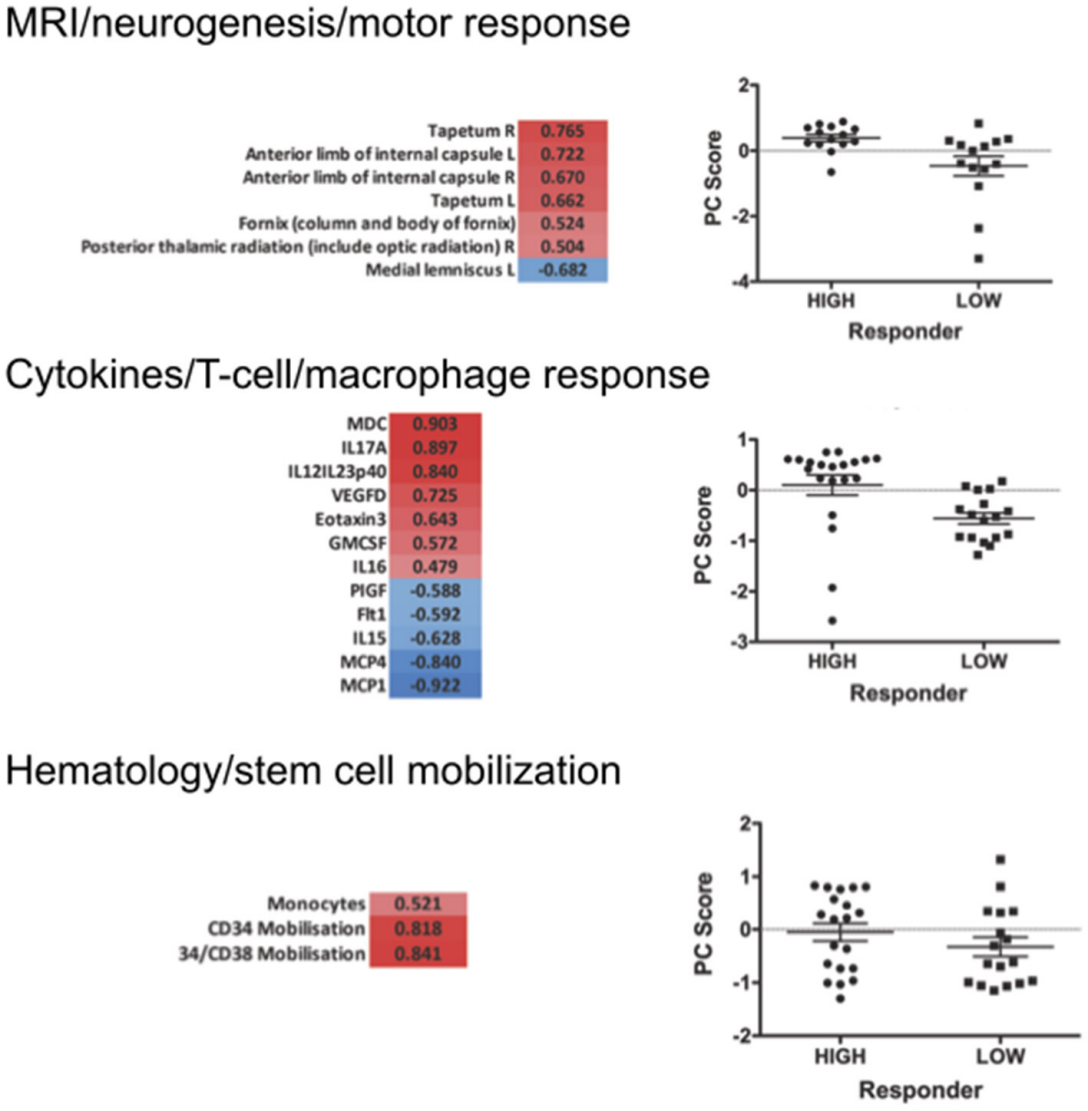
Selected PCA at 12 months of filgrastim treatment. Focusing upon selected non-linear PCA of neuroimaging (neurogenesis and motor response; first part), cytokines (T-cell and macrophage response; second), and hematology (stem cell mobilization; last part) at 12 months of treatment. The PC compounds explaining most of the variance were selected, and only items loading over a threshold of >0.5 were included to the analysis: PC3 reflecting motor system and neurogenesis was chosen for the imaging evaluation (*p* < 0.01); PC1 was selected for the cytokine data—reflecting the T-lymphocyte-macrophage response (*p* < 0.01); in hematology PC3 was selected, reflecting stem cell mobilization and monocyte mobilization (*p* < 0.01). The graphs give a comparison of scores on the selected PCs in responding vs. non-responding filgrastim patients. PCAs at 3 and 6 months are displayed in Supplementary Material.

**Figure 8:**
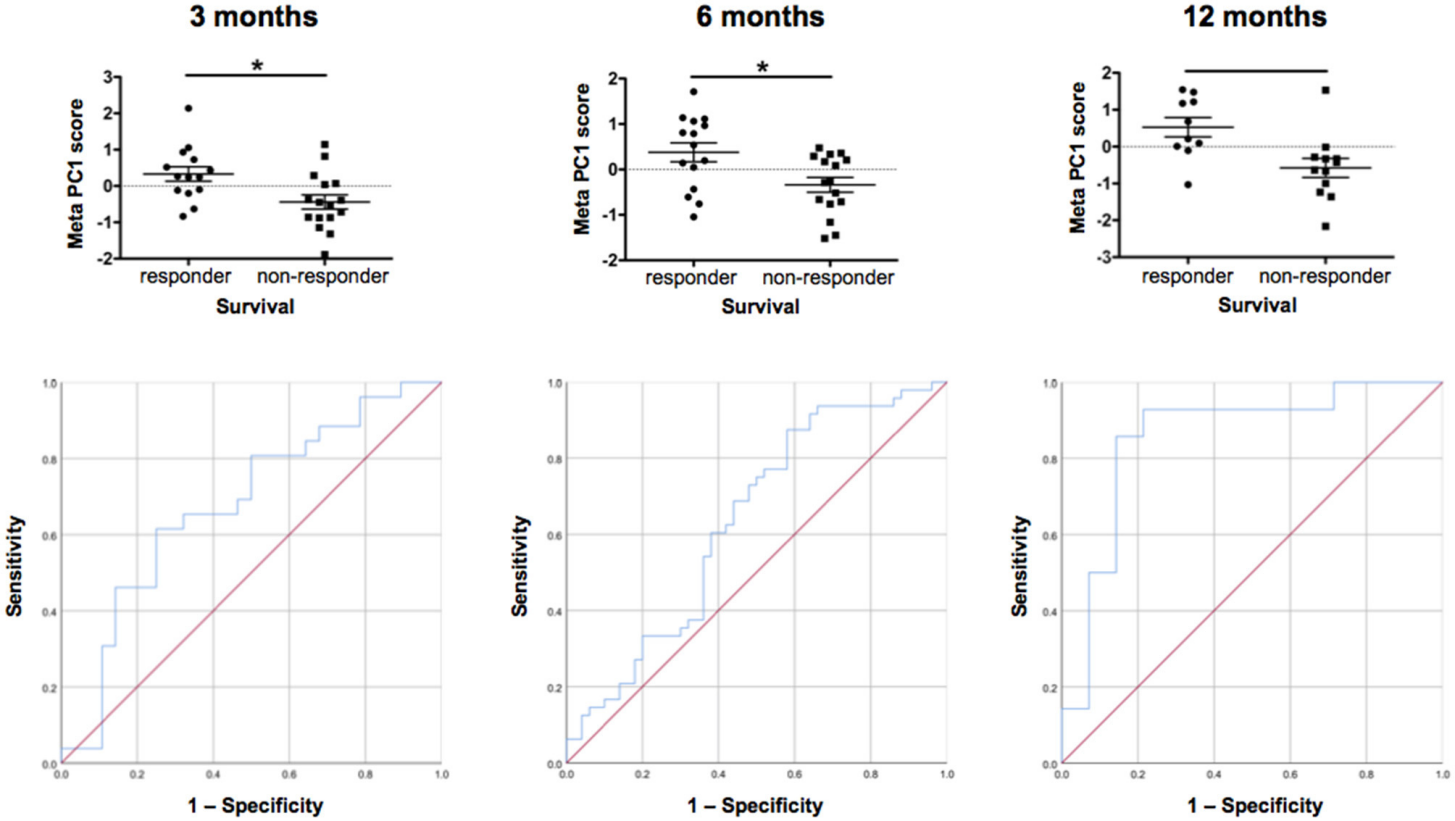
Individual patient response. Comparison of individual patient response by meta-PCs and testing of ROC (sensitivity/specificity) at 3, 6, and 12 months. We generated meta-PCs from the significant PCs at 3, 6, and 12 months of filgrastim treatment and analyzed the relationship between the meta-PCs and individual treatment response. Responding and non-responding patients have significantly different mean meta-PC scores at all timepoints, with patients responding better to treatment exhibiting higher scores (LMM, all *p* < 0.01). The receiver operating characteristic (ROC) curves showed that the meta-PCs were significantly predictive of whether patients would have responded to treatment or not (at 3 months: AUC = 0.677, *p* = 0.026; at 6 months: AUC = 0.633, *p* = 0.023; at 1 year: AUC = 0.854, *p* < 0.01). Although there is already quite a good prediction at 3 months, this will be much more accurate at 12 months. *p < 0.005.

We then clustered patients by treatment response (15 patients in each group) to analyze peripheral cytokines by an area under the curve (AUC) approach. Assessing timepoints at 3, 6, and 9 months of treatment revealed significantly higher serum levels of IL 6, MCP 1, eotaxin, and MCP 4, as well as TNF beta and IL 7 as a trend, in non-responding patients (Figure 5). We then assessed biomarker signatures in the treatment response groups by principal component (PC) analysis. After normalization for dimensionality, biomarkers from 36 filgrastim patients contained immune-biomarkers, hematology pre- and post-mobilization parameters, and follow-up data on 48 ROIs from DTI-based 1.5-Tesla MRI datasets (Figure 6). We selected the respective PC accounting for the highest percent of variance between patients within each biomarker package (imaging, hematology, and cytokines) at 3, 6, and 12 months of treatment (Figure 7, Supplementary Figures 9, 10). The correlation between each item (variable, e.g., specific cytokine) and the variance explained within this PC was determined as “loading” of that specific cytokine and used for ranking: only variables with an absolute loading threshold of >0.5 were included in the subsequent analysis. To assess the predictive validity of the biomarker packages, we compared individual patient scores on the selected PCs with response to filgrastim. Subsequently, we prepared a meta-PC analysis of only the significant PCs from individual clusters (Figure 8). For this, each patient was associated with a specific response PC score. This sensitivity/specificity analysis was executed by receiver operating characteristics (ROC) curves and applied separately at all given timepoints.

We first investigated the neuroimaging data at 12 months. PC3 was driven mainly by areas from motor system and neurogenesis and accounts for 9.5% of variance. Responding patients had significantly higher PC3 scores, indicative of more sustained motor system/neurogenesis than non-responders (*p* < 0.01; Figure 7). Hematology PC3 was driven by stem cell mobilization and monocytes (15.3% of the variance). Responders had significantly higher PC3 scores than non-responders (*p* < 0.01; Figure 7). In cytokine PC1, consisting mainly of inflammatory markers (23.9% of the variance), responding patients had a significantly higher loading (*p* < 0.01; Figure 7). From these significant PCs at 12 months, a meta-PC was generated, discriminating between responding and non-responding patients, with responders exhibiting higher scores (*p* < 0.01; Figure 8). ROC analysis confirmed the meta-PC at 1 year to be a significant predictor of patient response to treatment [area under the curve (AUC) = 0.854, *p* < 0.01].

Imaging data could not be analyzed at 6 and 3 months due to missing data. At 6 months of treatment, again hematology (PC1, 24.7% of the variance, *p* < 0.01) and stem cell markers (PC3, 12.9% of the variance, *p* < 0.019) as well as markers for inflammatory cytokines (PC1, 39.1% of the variance, and PC3, 14.4% of the variance, both *p* < 0.01) explained the variance in responding and non-responding patients (Supplementary Figure 10). This resulted in a more complex meta-PC, with responders exhibiting higher scores (*p* < 0.01; Figure 8). The corresponding ROC analysis confirms that the meta-PC at 6 months was modestly predictive of treatment response (AUC = 0.633, *p* = 0.023; Figure 8). At 3 months (Supplementary Figure 9), responders had higher scores in hematology PC3 (stem cells and monocytes, 13.6% of the variance, *p* < 0.01). For cytokines, two significant PCs separated the patient groups: responders had lower scores in PC1 (microvascular/macrophage, 25.8% of the variance) and higher scores in PC3 (T-cell/macrophage response, 15.5% of the variance) (both *p* < 0.01). The meta-PC was significantly predictive (ROC analysis) of treatment response (AUC = 0.677, *p* = 0.026; Figure 8). Consequently, a significant prediction of response was possible after only 3 months of treatment.

## Discussion

We retrospectively analyzed a novel therapeutic approach in ALS with long-term filgrastim application in 36 patients. We chose the well-known PRO-ACT database for reference and modeling. The treatment of a small number of ALS patients and the use of historical controls are obvious limitations one has to keep in mind when generalizing our results. To ensure comparability at baseline, we characterized the cohorts of filgrastim-treated and PRO-ACT patients, addressed the main outcome covariates, and adjusted for baseline difference whenever necessary. The comparability was quite adequate; however, filgrastim patients were on average 6 years younger than PRO-ACT patients, which was compensated for whenever needed (Table 1). The riluzole- and placebo-treated patients (rp-PRO-ACT) were the best-performing PRO-ACT cohort (Figure 1A) and most suitable comparators. The Cox proportional-hazard model gave a 0.52 hazard ratio for death in filgrastim vs. rp-PRO-ACT patients (Table 2). Matched-pair analysis (Supplementary Figure 3) and the AFT model (Figure 1B) confirmed this effect level in filgrastim therapy. This was clinically mirrored by analysis of ALSFRS-R progression at six (Supplementary Figure 4) and 36 months (Supplementary Figure 5). Interestingly, effects become more pronounced with shorter treatment latency (Supplementary Figure 6) and longer treatment duration—both to be expected in a CNS repair strategy.

A main achievement by our statistical approach was the generation of a model for survival estimation that was based on deceased patients in the PRO-ACT database. When this survival model was applied to filgrastim-treated patients, it classified them as responders or non-responders due to individual differences between model-estimated and observed survival time (Figure 3). The clinical relevance of the model-identified response classification was mirrored by less functional decline and impressively longer survival in filgrastim-responding patients (Figures 4A–C). In a next step, multimodal biomarkers were analyzed in the context of model-identified treatment response. This allowed an early and biomarker-driven identification of subsequent individual patient's treatment response. Here, treatment response was explicitly associated with early bioinformatic PCA patterns of multimodal structural, stem cell, and immune biomarkers: filgrastim responders mobilized stem cells more efficiently, had less inflammatory reaction, and showed relatively preserved cerebral white matter integrity (Figures 6–8). Altogether, this procedure not only shed light on possible modes of action of filgrastim in ALS but may also pave the methodological way for other therapeutic options in ALS patients.

As we did not perform genetic testing, lack of information on genetic background is a limitation when analyzing the data. We found that younger filgrastim patients responded better to treatment; however, on top of their age-related prognostic advantage, they survived considerably longer than their age-matched PRO-ACT counterparts (Supplementary Figure 8, Figure 4D). With filgrastim, an age-dependent benefit is plausible. Direct CNS effects as well as indirect effects mediated by hematopoietic stem cells have been described (4). Both CNS and the hematopoietic system suffer from age-dependent decline of regenerative capacities. Elderly individuals mobilize hematopoietic stem cells less efficiently (23) and exhibit an elevated inflammatory response (24). In stem cell transplantation, younger age is the only donor factor associated with longer receiver survival (25). A 60-year limit is accepted in filgrastim mobilization for stem cell harvesting^2^. However, our model revealed that also some elderly filgrastim patients lived longer than predicted—the individual stem cell age and immune functions seem to be crucial, as shown in a recent paper (9). Being aware of our non-randomized experimental setting and small patient number, our data are the first to show that drug-mobilized autologous stem cells have a significant impact on performance and survival in a neurodegenerative disorder like ALS (19).

Filgrastim may exert beneficial effects in ALS by multiple possible mechanisms of action. It is a neuronal growth factor within the CNS that exerts neuroprotective properties (3, 4) and induces neurogenesis (3, 8, 26). Specifically, neuroinflammation is increasingly recognized in ALS pathogenesis (2), and immune modulating effects by filgrastim may establish a more protective immune status (27). Filgrastim mobilizes hematopoietic stem cells that may migrate to the CNS and may offer trophic support and immune modulation within the CNS (28, 29). Therefore, the responding and non-responding ALS patients were characterized by peripheral cytokines. Patients responding to treatment had indeed lower levels of inflammatory cytokines over the first 9 months of treatment (Figure 5).

The impact of a biomarker-guided individualized ALS therapy was highlighted by modeling. We addressed the complexity of individual ALS patients by modeling the different pathophysiologic impacts of inflammation, stem cells, and brain structure, as well as biomarker response to treatment, in a comprehensive way, not paralleled before in ALS. We thereby detected approximately 40% of strong filgrastim responders. We need to keep in mind that in other patient populations response rates may be higher or lower, depending on individual compositions, treatment latency, and duration. Indeed, biomarker evaluation allowed assignment of patient response to filgrastim as early as within 3 months of treatment. Thus, treatment time windows for response known from oncology may also apply for neurodegeneration; this strongly highlights the imperative necessity of early therapeutic intervention.

Filgrastim is a widely used, well-tolerated drug, with an excellent safety profile in ALS that has been highlighted by Wallner et al. (4) and our group (9). Minor adverse events were mild to moderate bone pain and—as expected—leukocytosis after injection. We found that filgrastim-treated patients survived longer than PRO-ACT patients on a group level, but also longer than individually predicted by modeling. This further supports filgrastim as a safe therapeutic option in ALS. Earlier filgrastim studies in ALS patients have displayed positive effects on inflammation and neuroimaging (11, 12), but the promising survival data from animal models have not yet been translated to patients. In comparison to earlier studies in ALS patients (4), our positive outcome may have been achieved by higher doses of filgrastim, in part shorter treatment latencies, more frequent applications, and by far more extended treatment duration.

In this diligent and in-depth comparison of survival and functional decline in filgrastim-treated patients, we considered all PRO-ACT patients as reference and then narrowed the comparison down to the most suitable cohort of rp-PRO-ACT patients, which was also used for further modeling. We paid particular attention to relevant methodical bias, made adjustments for demographic and clinical differences between the two groups, and conducted analysis in carefully matched patients. Comparable results in different statistical approaches confirmed the stability of the difference in functional decline and survival between filgrastim and PRO-ACT. We developed a PRO-ACT-based model for individual survival prediction and applied this model to filgrastim patients to detect treatment response. Individuals most responsive to treatment were then identified at the early stage by principal component biomarker analysis for stem cells, neuroinflammation, and structural imaging. Although the treatment effect is impressively associated with age—young patients benefited most from treatment—also some of the elder patients were clearly treatment responders. Subcutaneous filgrastim has a favorable safety profile: thus, the benefit risk ratio is positive, as our data give a clear signal of efficacy with both slower clinical progression and increased survival in ALS patients.

## Data Availability Statement

The raw data supporting the conclusions of this article will be made available by the authors, without undue reservation.

## Ethics Statement

The studies involving human participants were reviewed and approved by Ethics committee of the University of Regensburg, Regensburg, Germany. The patients/participants provided their written informed consent to participate in this study.

## Author Contributions

SJ: care for ALS patients, conception of intervention, acquisition, analysis, interpretation of data, and wrote the manuscript. JH, WK, and AF: substantial contribution to the conception of the study analysis, interpretation of data, and revision of the manuscript. BB and AS: substantial contribution to the conception of the study, analysis, interpretation of data, and revision of the manuscript. SP, EW, and SK: performed cytokine experiments and revision of the manuscript. AW: acquisition and analysis of MRI data and revision of the manuscript. SI and JG: performed and analyzed HSPC experiments and revision of the manuscript. TK: organization of care and disposition of patient material. IK: assistance in maintenance of the database. MT and GS: acquisition of the MRI data and revision of the manuscript. TP: revision of the manuscript. LA: discussion, critical review of intervention and analysis, and revision of the manuscript. WS-M: care for ALS patients, conception of intervention and analysis, and revision of the manuscript. T-HB: conception of intervention and analysis, and revision of the manuscript. UB: care for ALS patients, conception of intervention and analysis, revision of the manuscript, and clinical responsibility for intervention. All authors contributed to the article and approved the submitted version.

## Conflict of Interest

UB and LA hold patents for clinical application of G-CSF in ALS. Orphan Drug Status is granted for EU and US by EMA and FDA—all within NeuroVision Pharma GmbH, Murnau, Germany. UB, LA, and T-HB are owners of the company Velvio GmbH. WK is the owner of the company BDS Koch. The remaining authors declare that the research was conducted in the absence of any commercial or financial relationships that could be construed as a potential conflict of interest.

## Footnotes

^1^Available online at: https://nctu.partners.org/ProACT/Data/Index/1nctu.partners.org

^2^Available online at: https://bethematch.org/transplant-basics/matching-patients-with-donors/why-donor-age-matters/

## Supplementary Material

Supplementary Figure 1 Illustration of individual filgrastim treatment evolving over time. ALS patients were seen at the outpatient clinic once or twice á month with assessment of clinical status and laboratory work-up. Further, once to twice á month hematopoietic stem cells were assessed in peripheral blood and blood samples were stored for later evaluation of cytokines and chemokines. The duration of individual filgrastim treatment is given by the framed area, the color intensity indicates the mean monthly filgrastim dose, which is also specified in Mio IU/month. The gray bars show survival after filgrastim treatment was ended, “>” gives time of death, and “I” the end of data entry. Filgrastim application modes were adapted over time. Filgrastim was mainly given either in intervals **(A–D)** or continuously on single days [**(E–G)**, and “other”]. **(A)** Filgrastim once or twice á day over 5 following days á month. (B) as in “A” with repetition of interval once á month (i.e., 2 × 5 days). **(C)** as in “B” with additional application every second day between intervals. **(D)** Three days of application á month. **(E)** Application once or twice á day on a single day á week. **(F)** Application every second day. [**(G)** and “other”]: different combinations of applications on single days á week. Filgrastim treatment was terminated in patient number 21. After 39 months of application he experienced an episode of heat sensation, lightheadedness, and 15 min of dyspnea. As drug-related intolerance or mild allergic reaction could not be excluded, filgrastim was ended in this patient. Antibodies against filgrastim were not detected. This patient was switched to PEGylated G-CSF (Pegfilgrastim 6 mg) from his 46th to 53rd month of treatment and then ended the treatment without further adverse reactions.

Supplementary Figure 2 Adjusted Cox Proportional Hazards fit and parametric survival analyses with all available PRO-ACT patients. Significance of survival contribution of a factor is given by it's *p*-value. The significant term “database” refers to filgrastim or PRO-ACT and indicates treatment effect. Number of events 1,280; number of censorings 264; total number 1,544. The model predicted adjusted increase in median survival time under filgrastim compared to PRO-ACT was 11.4 months (*p* < 0.0001) while the increase in model predicted median survival time under riluzole (yes vs. no) was about 0.7 months only (*p* = 0.5351).

Supplementary Figure 3 Matched pairs, YP-model. Significant survival differences are given by p-values from 10,000 repeats of matching each filgrastim patient to 10 PRO-ACT patients that were selected from both all PRO-ACT patients (first graph) as well as only patients from the rp-PRO-ACT subgroup (second graph) by YP-model. Mean survival in filgrastim treated patients was significantly longer (596 days), compared to the PRO-ACT matched patients (373 days; survival given as mean of the medians in all 100,000 draws; all *p* < 0.001 by YP test). Comparison of filgrastim (596 days) with the matched patients from the rp-PRO-ACT subgroup revealed a significant difference with survival at 403 days in this group (median *p*-value of 0.005, range of *p*-values: 0.0001–0.07, by YP test).

Supplementary Figure 4 Table of all fixed effect variables included into the mixed effect model for estimation of ALSFRS-R decline over the first 6 months. “Database” refers to the two groups of filgrastim treated vs. PRO-ACT patients treated with riluzole. The significant interaction term “database*month” indicates a different steepness in functional decline depending on treatment group. The effect variables are sorted by increasing *p*-value (last column).

Supplementary Figure 5 Visualization of individual ALSFRS-R data points over 6 months. Individual ALSFRS-R scores (dots) over the first 6 months: first graph (green): *N* = 36 filgrastim treated patients, second graph (blue): *N* = 6,927 riluzole receiving PRO-ACT patients. Individual data points and smoothing splines visualized the ALSFRS-R scores with 95%-CIs.

Supplementary Figure 6 Table of all fixed effect variables included into the mixed effect model for estimation of ALSFRS-R decline over the first 6 months in patients with treatment latency shorter than 10 months. “Database” refers to the two groups of filgrastim treated vs. all PRO-ACT patients from only newer studies. The significant interaction term “database*month” indicates a different steepness in functional decline depending on treatment group. The effect variables are sorted by increasing *p*-value (last column).

Supplementary Figure 7 Table of all fixed effect variables included into the mixed effect model for estimation of ALSFRS-R. “Responder group” refers to the two groups of filgrastim treated, who have a longer or shorter survival than model-estimated. The significant interaction term “month*responder group” indicates a different steepness in functional decline depending on treatment group. The effect variables are sorted by increasing *p*-value (last column).

Supplementary Figure 8 Parametric survival model with interaction term. The contributing factors to the model for survival estimation are given. The model was extended by the interaction term database*age. This interaction term captures a possible dependency between database (i.e., filgrastim or rp-PRO-ACT) and age.

Supplementary Figure 9 Selected PCA at 3 months. Selected non-linear PCA of cytokines (T-cell and macrophage response, **(A)** microvascular and macrophage response, **(B)** and stem cell mobilization **(C)** at 3 months of treatment. The PC compounds explaining most of the variance were selected, and only items loading over a threshold of >0.5 were included to the analysis. The graphs give a comparison of scores on the selected PCs in responding vs. non-responding filgrastim patients.

Supplementary Figure 10 Selected PCA at six months. Selected non-linear PCA of cytokines (T-cell and macrophage response **(A)**, Killer-cell and macrophage response **(B)**, stem cell mobilization **(C)**, as well as hematopoietic response **(D)** at 6 months of treatment. The PC compounds explaining most of the variance were selected, and only items loading over a threshold of >0.5 were included to the analysis. The graphs give a comparison of scores on the selected PCs in responding versus non-responding filgrastim patients.
