# Supplementary material for: Modeling and Bioinformatics Identify Responders to G-CSF in Patients With Amyotrophic Lateral Sclerosis
Source: Front Neurol. 2021 Mar 18;12:616289. doi: 10.3389/fneur.2021.616289 (PMC8012841; doi:10.3389/fneur.2021.616289)
Supplement: Supplementary Figure 1 — Illustration of individual filgrastim treatment evolving over time. ALS patients were seen at the outpatient clinic once or twice á month with assessment of clinical status and laboratory work-up. Further, once to twice á month hematopoietic stem cells were assessed in peripheral blood and blood samples were stored for later evaluation of cytokines and chemokines. The duration of individual filgrastim treatment is given by the framed area, the color intensity indicates the mean monthly filgrastim dose, which is also specified in Mio IU/month. The gray bars show survival after filgrastim treatment was ended, “>” gives time of death, and “I” the end of data entry. Filgrastim application modes were adapted over time. Filgrastim was mainly given either in intervals (A–D) or continuously on single days [(E–G), and “other”]. (A) Filgrastim once or twice á day over 5 following days á month. (B) as in “A” with repetition of interval once á month (i.e., 2 × 5 days). (C) as in “B” with additional application every second day between intervals. (D) Three days of application á month. (E) Application once or twice á day on a single day á week. (F) Application every second day. [(G) and “other”]: different combinations of applications on single days á week. Filgrastim treatment was terminated in patient number 21. After 39 months of application he experienced an episode of heat sensation, lightheadedness, and 15 min of dyspnea. As drug-related intolerance or mild allergic reaction could not be excluded, filgrastim was ended in this patient. Antibodies against filgrastim were not detected. This patient was switched to PEGylated G-CSF (Pegfilgrastim 6 mg) from his 46th to 53rd month of treatment and then ended the treatment without further adverse reactions. [file Data_Sheet_1.docx]

Supplementary Material

# Supplementary Methods

## Description of filgrastim treated patients and intervention

As this was not a prospective clinical trial and with the intention to match the real-world situation, the use of formal exclusion criteria was not considered appropriate. However, neither patients with a current or past history of neurologic disease other than ALS, nor patients participating in any interventional study were offered this treatment option. The principles of the Declaration of Helsinki (World Medical Association, revised version 2013) were strictly adhered to. From January 2010 on, 36 ALS patients were treated with subcutaneous injections of filgrastim. Patients were seen on a monthly basis with clinical examinations, monitoring of ALS progression by ALSFRS-R *(33)*, analysis of blood counts, cytokine expressions, and estimation of bone marrow function. We conducted cerebral DTI-based MRI at baseline and over time. Survival was defined as the time between treatment initiation and death, regardless of the cause. Detailed information on treatment and obtained clinical and laboratory measurements has been published previously *(9)*.

## Description of PRO-ACT, statistical analysis plan and cohort comparability

The PRO-ACT database was used as control dataset for survival and functional decline in filgrastim treated patients. PRO-ACT has amongst others been used in endpoint comparison for clinical trials *(26)*, for building predictions of functional decline and survival *(27)*, for patient subgroup stratification *(31)*, and for virtual trial conduction *(34)*. PRO-ACT databases in the versions from 2014 and 2016 were available. Analyses were carried out with the latest version. PRO-ACT data were imported from excel into JMP version 12 (SAS Institute). The following corrections or changes were made to the original PRO-ACT dataset: 1) If only ALSFRS, and no revised values (ALSFRS-R) were present, then the revised values were imputed from ALSFRS values by addition of eight. This was derived from comparing ALSFRS and ALSFRS-R values in PRO-ACT where both values were available. 2) If missing, ALSFRS-R values at baseline were derived from the closest value to time point of treatment start within a range of +/- 60 days. Values left of zero were preferred if two equal-distanced data points were available. If data were missing within this time range, then patients were removed from the analyses on ALSFRS-R decline. In survival analyses for patients that had missing values in ALSFRS-R baseline, following the previously described strategy, ALSFRS-R baselines were imputed as median values from the set of baselines present within the survival analysis set. 3) Patients with negative or zero survival times were not considered. 4) Entries of follow-up time points with no ALSFRS or ALSFRS-R value were removed (but patients kept). 5) The time point of survival censoring in non-deceased PRO-ACT patients was selected at the last given clinical visit with assessment of functional status. 6) To identify newer studies within PRO-ACT the attribute ’new patients’ was generated by entries of the revised ALSFRS-R values and by identification of patients only present in the 2016 database version. 7) Lastly, imputations were done as median as indicated in the specific analysis section.

Following our statistical analysis plan, analyses were carried out with both all available PRO-ACT patients, and different data subgroups of patients from recent studies only, riluzole-treated patients, and the group of riluzole- and placebo-treated PRO-ACT patients (“rp-PRO-ACT”). The comparability at baseline in the filgrastim patients and the PRO-ACT database was analyzed by standard descriptive statistical methods. Analyses were focused on patients with available survival data (survival data set). The PRO-ACT database contained survival/censoring data on 4658 patients. 41 of these patients had a follow-up time of zero and were thus excluded from survival analyses. The final survival dataset of 4617 patients contained complete information on survival/censoring, sex, and whether the study was of newer or older origin. Of these, 1544 patients (33.4%) also had complete data on age, site of onset, treatment latency, and ALSFRS-R. The remaining patients had one or more of these covariates missing. Baseline characteristics of the filgrastim treated patients and PRO-ACT treatment groups were compared by one-way ANOVA and illustrated by box-plot. The PRO-ACT groups were well comparable regarding age. As the mean difference in age between the filgrastim and the rp-PRO-ACT group was 6.49 years (p=0.0004, Student´s t-test), adjustment for age was required. Although the difference in functional status between groups was small and not statistically significant (p>0.05 for all comparisons by Student´s report-test), ALSFRS-R was used for adjustment and matching due to its high influence on survival. Further, although the mean differences between filgrastim and PRO-ACT in treatment latency, presumably an indicator of early progression speed, were not statistically significant, this variable was used as covariate in all modelling. The greatest difference in treatment latency (48 days) was found between two PRO-ACT groups (p<0.05, Student´s ordered differences report). As there were 10% less female patients in the filgrastim group, this variable was used for adjustment and matching. Limb onset of disease was observed in 83.3% of patients in the filgrastim group. This is somewhat more frequent than in the general ALS population where bulbar onset of disease occurs in 32% *(1)*. However, site of onset was missing for 54% of PRO-ACT patients (n=2493), and due to this lack of data, site of onset was not included in the main survival analyses. As there have been additions to the PRO-ACT database over time, we investigated the amount of newer patient data within the database. PRO-ACT does not allow access to individual trial information and identification of absolute time of treatment. Therefore, we identified proxies that allow a crude distinction into newer and older studies, namely emergence of ALSFRS-R as a revision of the functional scoring system *(33)*, and addition of studies performed at a later point in time as judged from the addition of patients from PRO-ACT version 2014 to 2016. The data from 79.9% of patients in the rp-PRO-ACT group were generated from newer studies, which further supports the good comparability of this study group to the filgrastim group. In the other two PRO-ACT groups, the percentage of patients in newer studies was considerably lower. Sensitivity analyses were then performed for patients in newer studies. Further, the filgrastim and the rp-PRO-ACT group have a comparable amount of patients with censored survival times (30.6% and 37.6% respectively).

## Survival analyses

The final PRO-ACT dataset used for survival analyses included 4617 patients as defined in the preceding section. We analyzed survival in the filgrastim treated group and in all patients within the PRO-ACT database, as well as the rp-PRO-ACT subgroup by non-adjusted Kaplan-Meier analysis. Further, the amount of deceased and censored patients in these groups was highlighted. The following variables were considered to adjust for differences of the filgrastim and PRO-ACT groups at baseline: age, sex, ALSFRS-R, site of onset (limb or bulbar), treatment latency (between disease onset and treatment initiation, termed ”onset delta days”) and use of riluzole. The availability of these factors and their respective influence on survival within PRO-ACT was further investigated. Database (filgrastim or PRO-ACT; both all PRO-ACT and the rp-PRO-ACT subgroup) was the variable of main interest in these adjusted comparisons. In addition, to explore a possible dependency of the filgrastim treatment effect from age, the preceding model was extended by the interaction term *database*age*.

The accelerated failure time (AFT) analysis was added as survival analysis, as this parametric model does not rely on assumption of proportional hazards. Survival was analyzed in filgrastim versus the rp-PRO-ACT subgroup, and described by hazard ratio and event time ratio (ETR). We used a Weibull accelerated failure time regression, performed in R using the ”survreg” function of package ”survival” (data not shown). The Weibull distribution was chosen as a good fit for our case, as the log-minus-log plot shows good approximation of a straight line. To obtain information on the ETR or acceleration and deceleration factors in time, we conducted an extended analysis using the function ”WeibullReg” from package ”SurvRegCensCov” *(35)*.

Finally, following a regulatory advice, we used a matched pairs approach with bootstrapping for an alternative approach on survival comparison. The R-packages optmatch and rcbalance were applied. By a bootstrapping approach with 10000 draws, each filgrastim-treated patient was matched with the patients from both the overall PRO-ACT cohort, and from the rp-PRO-ACT subgroup. For every filgrastim patient, then the ten best matching PRO-ACT patients, regarding sex, age and ALSFRS-R at baseline, were selected. Due to age difference, the matching for filgrastim patients with lower age showed a higher variance. Also, the matching for filgrastim patients with a lower ALSFRS-R at baseline showed a higher variance. We then applied a test for equivalence (TOST) *(36)* to verify the results from the visual screen. As the matching algorithm was found to be appropriate, we proceeded with the survival comparison. From the ten respective rp-PRO-ACT matching counterparts of each filgrastim patient, nine were randomly included to a survival analysis together with the filgrastim patients, and survival curves were analysed by the YP model *(32)*.

## Analysis of functional decline

We assessed differences in the clinical course of filgrastim treated patients and patients from the PRO-ACT database. From 61406 ALSFRS-R score records in 6880 patients, 478 measurements were excluded due to missing data on study day or because patients had one ALSFRS-R measurement only, resulting in a database with 60928 ALSFRS-R measurements of 6599 patients (including the 36 filgrastim patients). Remaining missing values of ALSFSR-R at baseline were imputed by median ALSFRS-R baseline values; an indicator variable for imputation was generated. The median number of ALSFRS-R measurements in filgrastim patients was 12.5 (range: 2 to 72) and in PRO-ACT was 10 (range: 2 to 38). Multiple linear regression models of ALSFR-R included variables described in the article. In addition, patient ID was included as a random effect to take account for repeated ALSFRS-R score measurements (mixed model). A statistically significant interaction term *“database*month”* captured a possible treatment effect evolving approximately linearly over time. As our knowledge indicated that the treatment effect of filgrastim may be dependent on treatment latency in a more complex way, we generated an extended model including the three-way interaction term *“database*month*onset delta”* (onset delta indicating treatment latency). We estimated the mean ALSFRS-R time course over the first six months in filgrastim treated patients and patients within the PRO-ACT database with an average time from onset of symptoms to treatment initiation of 10 months.

## Biomarker signature for individualized treatment response

The industry standard panel for cytokine assessment (V-PLEX Human Biomarker 40-Plex Kit, MesoScale Discovery®, Maryland, USA) has been validated in different immune related and non-immune diseases (manufacture’s information). Peripheral blood serum was collected during regular visits at the hospital and immediately stored at -20°C for cytokine assays. For each assay, 25 µl of serum samples were used and test carried out in duplicates, according to the manufacturer´s instructions.

For hematological parameters, we analyzed white blood cells including cell differentiation, platelet, red blood cell counts, and hemoglobin levels with an automatic cell counter (Sysmex®, Kobe, Japan). Peripheral blood CD34^+^ and CD34^+^CD38^-^ hematopoietic stem and progenitor cells (HSPC) were analyzed by flow cytometry as earlier described by our group *(10)*. In short, 1 ml donor blood was lysed in 9ml NH_4_Cl lysis buffer and cells were then stained for 30 minutes at 4°C with combinations of anti-CD45-FITC (clone HI30, BD Pharmingen, Franklin Lakes, NJ, USA), CD34-APC (clone 581, Biolegend, San Diego, CA, USA) and CD38-PE (clone HIT2, BioLegend) monoclonal antibodies. Analysis was performed on a Becton Dickinson CALIBUR flow cytometer (BD, East Rutherford, NJ, USA).

Structural MRI was conducted at two 1.5 Tesla clinical scanners (Aera, Sonata, Siemens Medical, Erlangen, Germany). Diffusion weighted imaging (DWI) data were acquired with single shot spin-echo echo-planar sequences. The initial diffusion sequence of six orientations (DWI6) (TR: 3500ms, TE: 83ms, FA: 90°, FOV: 230 x 230 mm, 5mm slice thickness, 20 axial slices, b-value: 1000 s/mm², three b0 images) was modified to 20 orientations (DWI20) (TR: 3500ms, TE: 83ms, FA: 90°, FOV: 230x230mm, 5mm slice thickness, 20 axial slices, b-value: 1000 s/mm², three b0 images) during data acquisition period. Possible biases caused by the change of DWI sequence were controlled for and corrected by a separate control analysis. MRI data were processed using Freesurfer software 5.3 (Martinos Center for biomedical imaging, Charlestown, MA) and Matlab (Release 2012, The MathWorks, Inc., Natick, Massachusetts, USA). DWI raw data were preprocessed and atlas-based regions of interest (ROIs) were registered to individual diffusion tensor imaging (DWI) space. Maps of DTI-based fractional anisotropy (FA) were estimated. The Johns Hopkins University (JHU) WM atlas by Mori et al. *(37)* defined 48 WM regions in Montreal Neurological Institute (MNI) DWI space (resolution: 2mm). Average DWI space was registered to individual DWI space by using linear and nonlinear registration tools (FLIRT, FNIRT). Registrations were visually inspected and carefully adapted if necessary. All 48 WM regions were transferred to the individual DWI space. Mean FA values for all regions were calculated across voxels in every individual patient. We investigated the fractional anisotropy of these 48 regions of interest in diffusion tensor imaging based cerebral MRI.

The ALS patients were seen at the outpatient clinic on a monthly basis. However, due to practical reasons, the exact time points of biomarker investigation had to be defined as time periods. Thus, baseline data on ALSFRS-R and MRI were obtained from the day of treatment initiation +/- 28 days. Baseline measures of blood counts, stem cell mobilization parameters and cytokines were only taken from data obtained before the first filgrastim application. The three-month time point included days 45 to 134, the six-month time point days 135 to 224, the nine-month time point days 225 to 314, and lastly the 12-month time point days 315 to 449. If patients visited more than once during these time periods, the day closest to the intended time point was selected.

Cytokines were analyzed in the filgrastim response groups by an area under the curve (AUC) approach. AUC for each biomarker calculated with the R-package flux *(38)*. Non-linear principal component analyses (NLPCA) were applied in the evaluation of neuroimaging, hematology, and cytokine biomarkers. Statistical analyses were performed using Statistical Package for Social Sciences version 24 (IBM, Inc. Chicago, IL, USA).

# Supplementary Figures and Tables

##

**Supplement 1. Illustration of individual filgrastim treatment evolving over time.** ALS patients were seen at the outpatient clinic once or twice á month with assessment of clinical status and laboratory work-up. Further, once to twice á month hematopoietic stem cells were assessed in peripheral blood and blood samples were stored for later evaluation of cytokines and chemokines. The duration of individual filgrastim treatment is given by the framed area, the color intensity indicates the mean monthly filgrastim dose, which is also specified in Mio IU/month. The grey bars show survival after filgrastim treatment was ended, “>” gives time of death, and “I” the end of data entry. Filgrastim application modes were adapted over time. Filgrastim was mainly given either in intervals (A, B, C, and D) or continuously on single days (E, F, G, and “other”):

A: Filgrastim once or twice á day over 5 following days á month. B: as in “A” with repetition of interval once á month (i.e. 2x 5 days). C: as in “B” with additional application every second day between intervals. D: Three days of application á month. E: Application once or twice á day on a single day á week. F: Application every second day. G and “other”: different combinations of applications on single days á week.

Filgrastim treatment was terminated in patient number 21. After 39 months of application he experienced an episode of heat sensation, lightheadedness and 15 min. of dyspnea. As drug-related intolerance or mild allergic reaction could not be excluded, filgrastim was ended in this patient. Antibodies against filgrastim were not detected. This patient was switched to PEGylated G-CSF (Pegfilgrastim 6 mg) from his 46^th^ to 53^rd^ month of treatment and then ended the treatment without further adverse reactions.

| Variable | Cox PH Model  Wald Tests | |  | Parametric Model Wald Tests | |
| --- | --- | --- | --- | --- | --- |
|  | Wald Chi Square | p-value |  | Wald Chi Square | p-value |
| Age | 119.70 | <0.0001 |  | 135.12 | <0.001 |
| ALSFRS-R at baseline | 89.80 | <0.0001 |  | 87.77 | <0.001 |
| Database | 15.69 | <0.0001 |  | 36.51 | <0.001 |
| Treatment latency | 15.38 | <0.0001 |  | 12.99 | 0.0003 |
| Sex | 12.45 | 0.0004 |  | 16.13 | <0.001 |
| Site of onset | 13.13 | 0.0044 |  | 13.02 | 0.0046 |
| Riluzole use | 0.40 | 0.8206 |  | 0.86 | 0.6501 |

| Risk Ratio Database | Risk Ratio | Prob Chi Square | Upper 95% | Lower 95% |
| --- | --- | --- | --- | --- |
| PRO-ACT vs filgrastim | 2.44 | <0.0001 | 1.57 | 3.80 |
| Filgrastim vs PRO-ACT | 0.41 | <0.0001 | 0.26 | 0.64 |
| Risk Ratio  Riluzole use | Risk Ratio | Prob Chi Square | Upper 95% | Lower 95% |
| Yes vs no | 0.96 | 0.5351 | 0.85 | 1.09 |
| No vs yes | 1.04 | 0.5351 | 0.92 | 1.17 |

**Supplement 2. Adjusted Cox Proportional Hazards fit and parametric survival analyses** with all available PRO-ACT patients. Significance of survival contribution of a factor is given by it´s p-value. The significant term “database” refers to filgrastim or PRO-ACT and indicates treatment effect. Number of events 1280; number of censorings 264; total number 1544. The model predicted adjusted increase in median survival time under filgrastim compared to PRO-ACT was 11.4 months (p<0.0001) while the increase in model predicted median survival time under riluzole (yes versus no) was about 0.7 months only (p=0.5351).


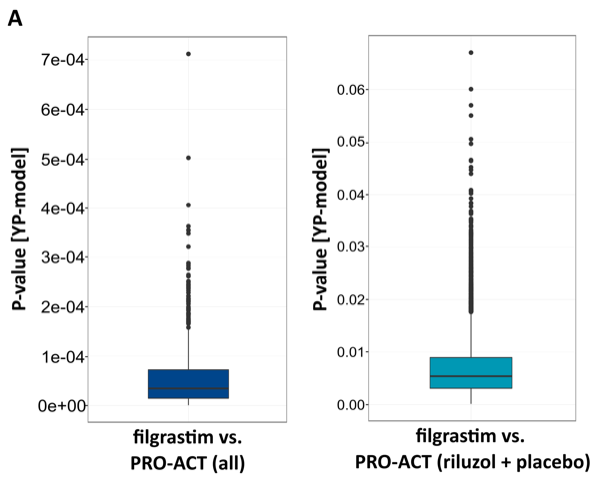


**Supplement 3. Matched pairs, YP-model.** Significant survival differences are given by p-values from 10000 repeats of matching each filgrastim patient to 10 PRO-ACT patients that were selected from both all PRO-ACT patients (first graph) as well as only patients from the rp-PRO-ACT subgroup (second graph) by YP-model. Mean survival in filgrastim treated patients was significantly longer (596 days), compared to the PRO-ACT matched patients (373 days; survival given as mean of the medians in all 100000 draws; all p-values <0.001 by YP test). Comparison of filgrastim (596 days) with the matched patients from the rp-PRO-ACT subgroup revealed a significant difference with survival at 403 days in this group (median p-value of 0.005, range of p-values: 0.0001 to 0.07, by YP test).

| Variable | DF | DFDen | F Ratio | p-value |
| --- | --- | --- | --- | --- |
| ALSFRS-R at baseline | 1 | 1636 | 8180,183 | <.0001 |
| Month and knotted | 2 | 5675 | 220,2448 | <.0001 |
| Treatment latency | 1 | 1542 | 133,4748 | <.0001 |
| Database*month | 1 | 5661 | 20,2853 | <.0001 |
| ALSFRS-R baseline Imp Ind | 1 | 1987 | 17,2220 | <.0001 |
| Sex | 1 | 1607 | 6,5529 | 0.0106 |
| Age | 1 | 1621 | 4,4845 | 0.0344 |
| Database | 1 | 1407 | 3,7057 | 0.0544 |

**Supplement 4.** **Table of all fixed effect variables** included into the mixed effect model for estimation of ALSFRS-R decline over the first six months. “Database” refers to the two groups of filgrastim treated versus PRO-ACT patients treated with riluzole. The significant interaction term “*database*month*” indicates a different steepness in functional decline depending on treatment group. The effect variables are sorted by increasing p-value (last column).


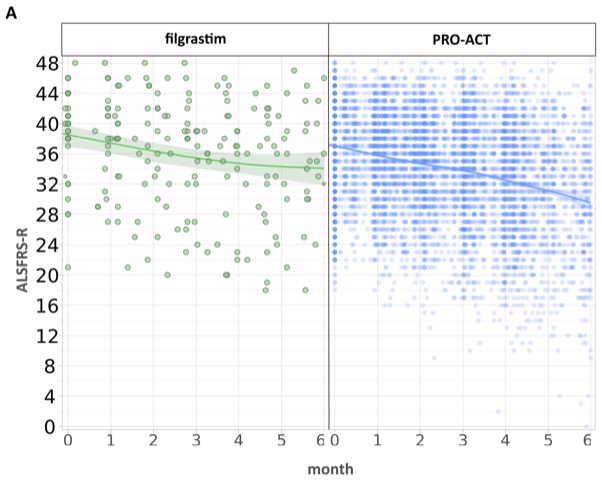


**Supplement 5. Visualization of individual ALSFRS-R data points over six months.** Individual ALSFRS-R scores (dots) over the first six months: first graph (green): N=36 filgrastim treated patients, second graph (blue): N=6927 riluzole receiving PRO-ACT patients. Individual data points and smoothing splines visualized the ALSFRS-R scores with 95%-CIs.

| Variable | Nparm | DF | DFDen | F Ratio | p-value |
| --- | --- | --- | --- | --- | --- |
| ALSFRS-R at baseline | 1 | 1 | 1174 | 5548,131 | <,0001 |
| Month and knotted | 2 | 2 | 3802 | 217,8286 | <,0001 |
| Log 10 treatment latency*month | 1 | 1 | 3780 | 33,5433 | <,0001 |
| Log10 treatment latency | 1 | 1 | 969,9 | 21,1754 | <,0001 |
| Database*month | 1 | 1 | 3793 | 20,6581 | <,0001 |
| Log10 treatment latency*database*month | 1 | 1 | 3780 | 13,5391 | 0,0002 |
| Age | 1 | 1 | 1137 | 5,4292 | 0,0200 |
| Sex | 1 | 1 | 1149 | 5,3584 | 0,0208 |
| Database | 1 | 1 | 968,3 | 3,7032 | 0,0546 |
| Log10 treatment latency*database | 1 | 1 | 966,1 | 1,0019 | 0,3171 |

**Supplement 6. Table of all fixed effect variables** included into the mixed effect model for estimation of ALSFRS-R decline over the first six months in patients with treatment latency shorter than 10 months. “Database” refers to the two groups of filgrastim treated versus all PRO-ACT patients from only newer studies. The significant interaction term “*database*month*” indicates a different steepness in functional decline depending on treatment group. The effect variables are sorted by increasing p-value (last column).

| Variable | Nparm | DF | DFDen | F Ratio | p-value |
| --- | --- | --- | --- | --- | --- |
| Month and knotted | 3 | 3 | 605.2 | 214.0162 | <.0001 |
| ALSFRS-R at baseline | 1 | 1 | 31.74 | 52.8496 | <.0001 |
| Month*responder group | 2 | 2 | 615.5 | 9.9159 | <.0001 |
| Responder group | 2 | 2 | 52.62 | 7.0071 | 0.0020 |
| Site of onset 4L | 1 | 1 | 31.59 | 5.0473 | 0.0318 |
| Sex | 1 | 1 | 33 | 2.0891 | 0.1578 |

**Supplement 7. Table of all fixed effect variables** included into the mixed effect model for estimation of ALSFRS-R. “Responder group” refers to the two groups of filgrastim treated, who have a longer or shorter survival than model-estimated. The significant interaction term “*month*responder group*” indicates a different steepness in functional decline depending on treatment group. The effect variables are sorted by increasing p-value (last column).

| Variable | Wald Chi Square | p-value |
| --- | --- | --- |
| ALSFRS-R at baseline | 40.68 | <.0001 |
| Age | 35.14 | <.0001 |
| Database | 8.97 | 0.0028 |
| Site of onset | 10.98 | 0.0118 |
| Sex | 5.83 | 0.0158 |
| Database*age | 4.50 | 0.0340 |
| Treatment latency | 4.25 | 0.0393 |

**Supplement 8.** **Parametric survival model with interaction term.** The contributing factors to the model for survival estimation are given. The model was extended by the interaction term *database*age*. This interaction term captures a possible dependency between database (i.e. filgrastim or rp-PRO-ACT) and age.


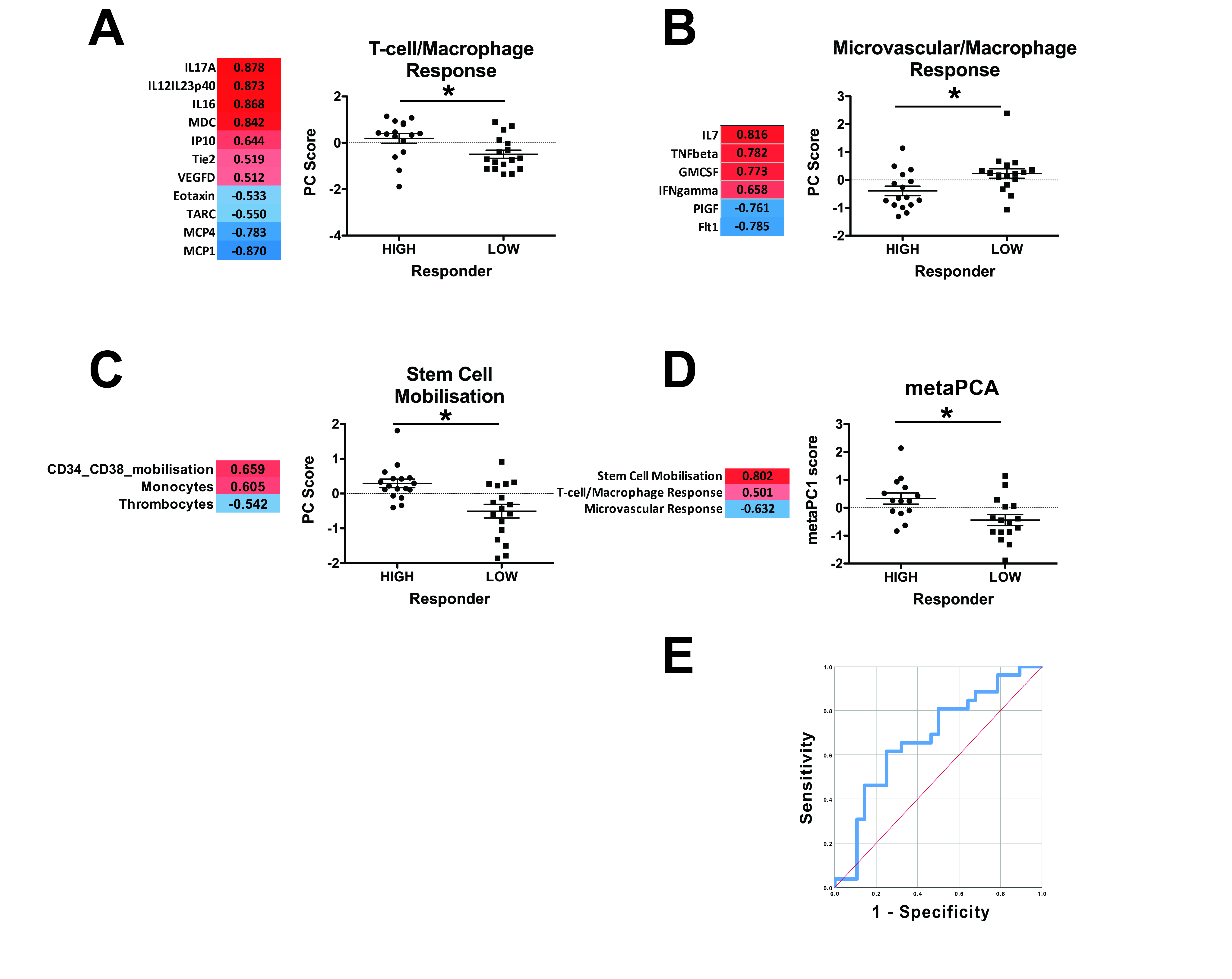


**Supplement 9. Selected PCA at three months.** Selected non-linear PCA of cytokines (T-cell and macrophage response, A; microvascular and macrophage response, B), and stem cell mobilization (C) at three months of treatment. The PC compounds explaining most of the variance were selected, and only items loading over a threshold of >0.5 were included to the analysis. The graphs give a comparison of scores on the selected PCs in responding versus non-responding filgrastim patients.


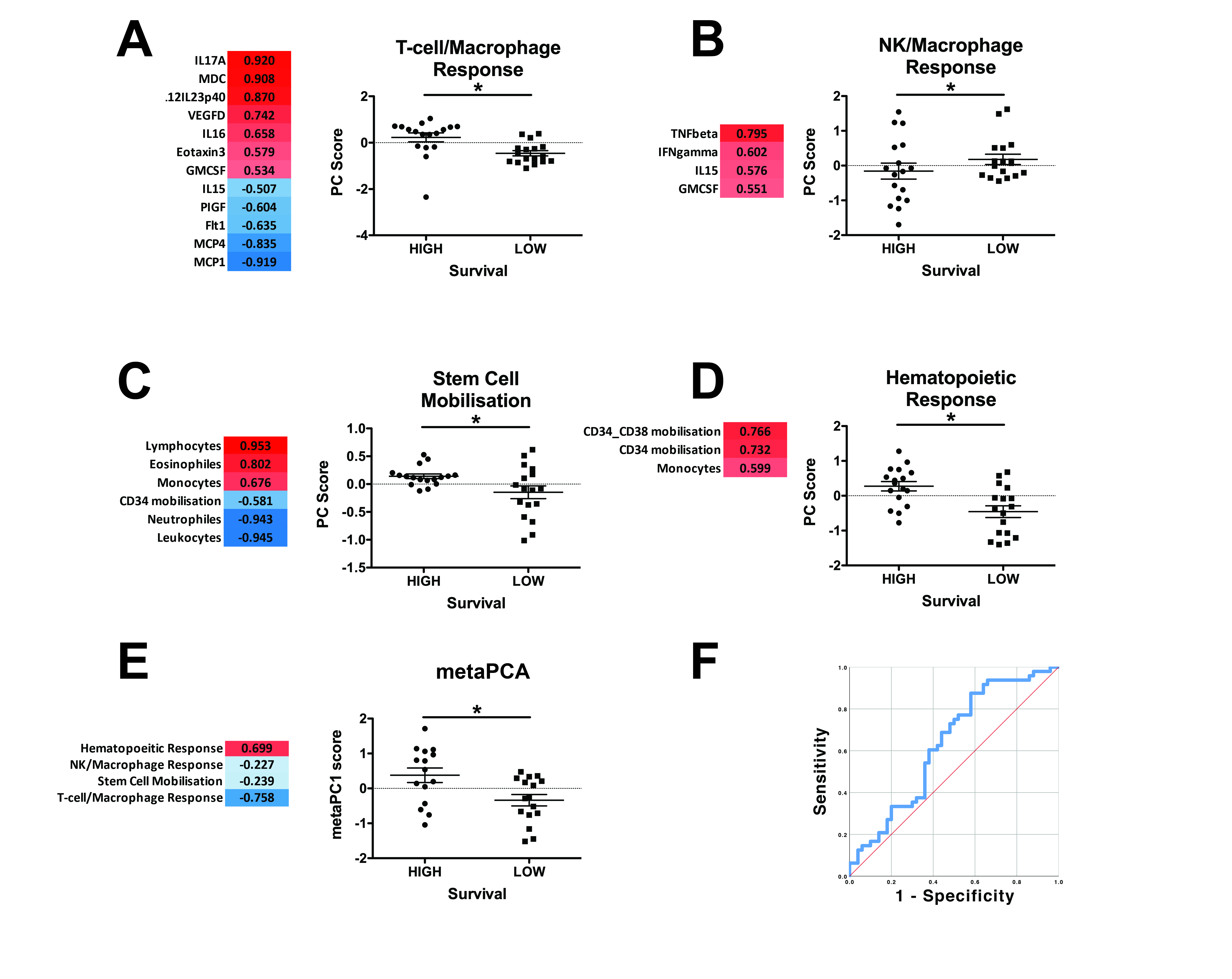


**Supplement 10. Selected PCA at six months.** Selected non-linear PCA of cytokines (T-cell and macrophage response, A; Killer-cell and macrophage responsem B), stem cell mobilization (C), as well as hematopoietic response (D) at six months of treatment. The PC compounds explaining most of the variance were selected, and only items loading over a threshold of >0.5 were included to the analysis. The graphs give a comparison of scores on the selected PCs in responding versus non-responding filgrastim patients.

**
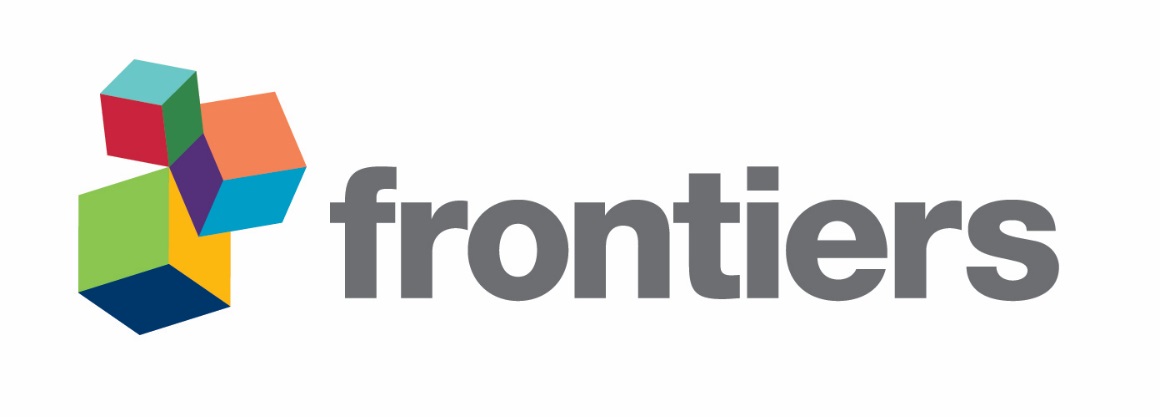
**
